# Knowledge and Use of PEP and PrEP Among Key Populations Tested in Community Centers in Portugal

**DOI:** 10.3389/fpubh.2021.673959

**Published:** 2021-07-23

**Authors:** Daniel Simões, Paula Meireles, Miguel Rocha, Rosa Freitas, Ana Aguiar, Henrique Barros

**Affiliations:** ^1^EPIUnit—Instituto de Saúde Pública, Universidade do Porto, Porto, Portugal; ^2^Grupo de Activistas em Tratamentos, Lisboa, Portugal

**Keywords:** HIV, pre-exposure prophylaxis, post-exposure prophylaxis, key population groups, community organizations

## Abstract

**Background:** Pre-exposure prophylaxis (PrEP) and post-exposure prophylaxis (PEP) have been increasingly available in Europe. Due to the high burden of HIV in key populations, these could benefit from their use. In 2016, in Portugal, an open, non-interval, prospective cohort study was established in a network of 26 community-based voluntary HIV/STI counseling and testing centers. Data collected included questions on PEP and PrEP knowledge and use. We aimed to estimate the proportion of PEP and PrEP knowledge and its use among key populations, visiting the centers between 2016 and 2019.

**Method and results:** Individuals who self-identify as being among at least one key population for HIV, men who have sex with men (MSM), people who inject drugs (PWID), sex workers (SW), migrants, and male-to-female transgender individuals (MTF), responded to questions on PEP and PrEP knowledge and use while waiting for their test results between 2016 and 2019 (*n* = 12,893 for PEP; *n* = 10,973 for PrEP). Reported knowledge was low in all key populations for both tools: 15.7% of respondents reported knowing about PEP and 10.9% about PrEP over the course of 4 years. PEP was used by 1.8% and PrEP by 0.4% of the respondents, MSM being 88.9% of PrEP users, and 52.8% of PEP users. Multivariate logistic regression showed multiple factors associated with knowing the tools, including age, education, country of birth, gender, year of test, having a reactive HIV test in the same visit, reporting an STI or condomless sex in the last 12 months, and identifying with being MSM or SW.

**Conclusions:** Knowledge and use of PEP and PrEP remain low among key populations in Portugal. The need remains to increase knowledge and use among those at risk for HIV infection.

## Introduction

In 2019, 136,449 new HIV infections were reported in the European Region of the WHO. Among the 59% with an established mode of transmission, heterosexual contact (50%), sex between men (24%), and injection drug use (15%) were the most reported. Among all cases with a known country of origin, 14% were migrants (1). In 2019, Portugal reported 778 new HIV infections, 55.7% being attributed to heterosexual transmission, 38% among men who have sex with men (MSM), and 2.1% among people who inject drugs (PWID). Migrants accounted for 37.7% of all cases with a known country of origin, 50.3% being reported in the Lisbon Metropolitan Area (2). Portugal has an HIV concentrated epidemic in both key populations and in urban areas, and notwithstanding 90–90–90 targets being met in 2018 (3), new HIV cases reveal missed opportunities in timely access and use of the available combination prevention tools, such as post-exposure prophylaxis (PEP) and pre-exposure prophylaxis (PrEP).

Post-exposure prophylaxis consists of a combination of antiretroviral (ARV) drugs taken by an HIV negative person for 28 days to prevent HIV seroconversion after a potential exposure in the last 48–72 h (4). Several countries have prescribed ARV as PEP since the 1990's, following an HIV occupational exposure (oPEP). This practice was gradually expanded to non-occupational situations/sexual intercourse (nPEP/PEPsi) (5). In Portugal, PEP has been available since the 1990's, limited to the National Health Service (NHS) hospitals, with emergency services being the only entry point to access this prevention tool (6). In 2008, nPEP/PEPsi was introduced in the NHS for serodiscordant couples through infectious disease outpatient clinics (7). Due to the absence of guidelines, each NHS hospital implemented its own protocol (8–11). Data on entry, uptake, and follow-up in the NHS hospitals of Portuguese PEP users are not published.

Human immunodeficiency virus PrEP consists of a combination of ARV drugs used by an HIV negative person older than 12 years old to prevent HIV seroconversion and taken before any potential HIV exposures (12, 13). The use of ARV as PrEP started in 2012, when the US Food and Drug Administration approved the use of tenofovir disoproxil fumarate and emtricitabine for HIV PrEP (14). The Portuguese PrEP recommendations were published in 2017 (15), and HIV PrEP has been available since 2018 at the hospital referral network for HIV infection (16–18). To NHS-registered users, irrespective of their legal status in the country, PrEP triage consultations, monitoring, and follow-up, as well as ARV provision, are free of charge (19). Data from November 2019 showed 1,000 PrEP users at the Portuguese NHS (3).

The objective of this study was to estimate the proportion of PEP and PrEP knowledge and use, as well as factors associated with the knowledge of both tools at the first test performed in one of the participating community-based organizations (CBOs), among five key populations: MSM, migrants, male-to-female transgender persons (MTF), sex workers (SW), and PWID who were tested in CBOs in Portugal between January 2016 and December 2019.

## Methods

Since 2016, 26 CBOs in Portugal have participated in the “Community-Based Screening Network” (Rede de Rastreio). These CBOs provide testing in a variety of settings, including fixed centers, mobile units, and outreach settings, that is, in locations where the key populations are. This includes specific neighborhoods, sex venues, drug use spaces, or migrant support centers, for example. All organizations target one or more key populations for HIV, and several include lay providers and peers of their target populations in their project teams.

Community-based organization staff received training and support in providing integrated testing for HIV, hepatitis C virus (HCV), hepatitis B virus (HBV), and syphilis. As part of the network, a standardized questionnaire was made available (online or paper version), enabling the collection of standardized data. Upon verbal consent, the participants provided information to generate a unique identifier, which allows linkage of subsequent visits, regardless of which network a person visits. This enabled the creation of a prospective cohort of people tested in the participating organizations, ongoing since January 2016.

The questionnaire includes social and demographic indicators (age, education level, place of birth, sex at birth, sexual orientation, and gender identity), testing history, reason(s) for testing, risk factors (condomless intercourse in the last 12 months, previous STI diagnosis in the last 12 months, engaging in commercial sex work, HBV vaccination status, previous piercings, tattoos or invasive medical procedures, and history of incarceration), drug use and sharing of drug use paraphernalia, knowledge and use of PEP and PrEP, and reported experience of violence.

Over the course of 4 years, a total of 53,809 baseline, 4,814 follow-up, and 7,020 refusal questionnaires were collected, which represent ~60,829 tested individuals (counting each refusal questionnaire as an individual). We analyzed the first completed questionnaires of people responding to at least one question of the PEP or PrEP sections of the questionnaire (*n* = 12,893 for PEP; *n* = 10,973 for PrEP) between January 2016 and December 2019 and those who identify as at least one of the following key populations:

- MSM—reported male sex at birth and gender and at least one male sexual partner in the last 12 months.
- SW—reported having had sex in exchange for money, goods, or services at any point in their lives.
- PWID—reported injectable drug use at any point in their lives.
- Migrant—reported country of birth other than Portugal.
- MTF—reported male sex at birth and female gender or reported gender identity as “male-to-female transgender.”

A Table with overall sample characteristics is available in Supplementary Table 1.

The participants were asked if they had ever heard of PEP and PrEP. Those who responded yes to each question were categorized as having knowledge of each prevention tool. Correct knowledge of PEP was defined as participants reporting: (a) PEP is a treatment to prevent an HIV infection; and (b) PEP has to be taken as quickly as possible following a potential HIV exposure. Correct knowledge of PrEP was defined as participants reporting: (a) PrEP is a tool to prevent an HIV infection and (b) PrEP has to be taken before a potential HIV exposure. The participants who reported affirmative to only one of these per tool were categorized as having incomplete knowledge. The participants that did not report affirmative to any of the two for each tool were categorized as having inappropriate knowledge of that tool.

Regions of birth were defined according to WHO Regions for Africa, Asia, and Oceania. South American and Caribbean countries were grouped in the same category (South America). Low HIV prevalence regions were grouped in one category, which includes Western Europe (except Portugal), United States of America, Canada, Oceania, and Middle East. The data were analyzed per key population as well as by selected variables: gender, age, region of birth, year of test, reporting diagnosis of sexually transmitted infection in the last 12 months, condomless intercourse in the last 12 months, reporting a previous HIV test, having an HIV reactive test at the baseline. The Chi squared or Fisher's exact test was used as appropriate. Crude and adjusted odds ratios (OR) and respective 95% CI were computed, using univariate and multivariate binomial logistic regressions. The statistical significance cutoff was.05. The data were analyzed in SPSS v24.0.

## Results

### Reported Knowledge of PEP and PrEP

Reported knowledge of both tools across the 4 years has remained low in all key populations (Tables 1, 2). Overall, 15.7% of the respondents were aware of PEP and 10.9% of PrEP. Reported knowledge of both tools increased over time (7.4% in 2016 to 16.3% in 2019 for PEP and 2.8% in 2016 to 16.4% in 2019 for PrEP; *p* < 0.001 for both) and varied across the different key populations.

**Table 1:** Number and percentage of respondents reporting to know PEP.

	**Does not know PEP**	**Knows PEP but never used**	**Knows PEP and used**	**Total**
Total	10,868 (84.3%)	1,794 (13.9%)	231 (1.8%)	0
**Gender**				***p*****-value**
Male	5,504 (82.3%)	1,037 (15.5%)	150 (2.2%)	<0.001
Female	5,293 (87.4%)	690 (11.4%)	71 (1.2%)	
MTF	64 (46.4%)	65 (47.1%)	9 (6.5%)	
FTM	7 (70.0%)	2 (20.0%)	1 (10.0%)	
**Age**				***p*****-value**
≤ 25 years	2„787 (83.5%)	500 (15.0%)	51 (1.5%)	<0.001
26–49 years	6,015 (81.6%)	1,182 (16.0%)	171 (2.3%)	
≥ 50 years	2,053 (94.5%)	111 (5.1%)	9 (0.4%)	
**Region of birth**				***p*****-value**
Portugal	2,672 (76.6%)	747 (21.4%)	70 (2.0%)	<0.001
Low prevalence regions (a)	816 (80.2%)	186 (18.3%)	15 (1.5%)	
South America	2,842 (78.7%)	644 (17.8%)	125 (3.5%)	
Africa	4,029 (95.5%)	173 (4.1%)	15 (0.4%)	
Eastern Europe and Asia	495 (91.2%)	42 (7.7%)	6 (1.1%)	
**Highest completed level of education**				***p*****-value**
Basic (≤ 9 years)	4,230 (93.3%)	288 (6.4%)	17 (0.4%)	<0.001
Secondary (12 years)	3,638 (82.9%)	656 (15.0%)	93 (2.1%)	
University (bachelors or higher)	2,554 (73.2%)	819 (23.5%)	117 (3.1%)	
**Test year**				***p*****-value**
2016	2,565 (92.7%)	185 (6.7%)	18 (0.7%)	<0.001
2017	1,898 (81.2%)	404 (17.3%)	35 (1.5%)	
2018	2,314 (79.9%)	510 (17.6%)	73 (2.5%)	
2019	4,091 (83.6%)	695 (14.2%)	105 (2.1%)	
**Key populations and other risk factors**				***p*****-value**
MSM (b)	1,383 (60.8%)	770 (33.8%)	122 (5.4%)	<0.001
PWID (b)	390 (85.5%)	57 (12.5%)	9 (2.0%)	0,595
SW (b)	1,787 (75.3%)	511 (21.5%)	75 (3.2%)	<0.001
Reported previous HIV test (b)	6,055 (77.9%)	1,494 (19.2%)	222 (2.9%)	<0.001
Reactive HIV test result on same visit (b)	156 (78.0%)	36 (18.0%)	8 (4.0%)	0,008
Reported STI last 12 months (b)	433 (73.0%)	138 (23.3%)	22 (3.7%)	<0.001
Reported condomless sex last 12 months (b)	8,172 (84.2%)	1,354 (14.0%)	176 (1.8%)	<0.001
**Knowledge of PrEP**				***p*****-value**
Reported knowing PrEP (b)	380 (27.6%)	863 (62.7%)	133 (9.7%)	<0.001

*PEP, post-exposure prophylaxis; MTF, male-to-female transgender; FTM, female-to-male transgender; MSM, men who have sex with men; SW, sex workers; PWID, people who inject drugs; STI, sexually transmitted infection*.

*(a)Low prevalence regions include Western Europe (except Portugal), the United States of America, Canada, Oceania, and the Middle East*.

*(b)Showing only results of the respondents reporting the respective category. P-value shown is relative to those not reporting the respective category/reporting the opposite of category shown*.

**Table 2:** Number and percentage of respondents reporting to know PrEP.

	**Does not know PrEP**	**Knows PrEP but never used**	**KnowsPrEP and used**	**Total**
Total	9580 (74.3%)	1348 (10.5%)	45 (0.4%)	10 973
**Gender**				***p*****-value**
Male	4,779 (82.0%)	1,013 (17.4%)	38 (0.7%)	<0.001
Female	4,721 (94.3%)	280 (5.6%)	4 (0.1%)	
MTF	73 (56.6%)	53 (41.1%)	3 (2.3%)	
FTM	7 (77.8%)	2 (22.2%)	0 (0.0%)	
**Age**				***p*****-value**
≤ 25 years	2,421 (83.8%)	458 (15.9%)	10 (0.3%)	
26–49 years	5,367 (86.1%)	830 (13.3%)	34 (0.5%)	
≥ 50 years	1,779 (96.7%)	59 (3.2%)	1 (0.1%)	
**Region of birth**				***p*****-value**
Portugal	2,502 (80.0%)	613 (19.6)	14 (0.4%)	<0.001
Low prevalence regions (a)	696 (83.5%)	136 (16.3%)	2 (0.2%)	
South America	2,618 (83.5%)	488 (15.6%)	28 (0.9%)	
Africa	3,329 (97.7%)	77 (2.3%)	0 (0.0%)	
Eastern Europe and Asia	420 (92.3%)	34 (7.5%)	1 (0.2%)	
**Highest completed level of education**				***p*****-value**
Basic (≤ 9 years)	3,684 (96.1%)	147 (3.8%)	2 (0.1%)	<0.001
Secondary (12 years)	3,222 (86.2%)	497 (13.3%)	20 (0.5%)	
University (Bachelors or higher)	2,285 (76.4%)	684 (22.9%)	21 (0.7%)	
**Test year**				***p*****-value**
2016	2,559 (97.3%)	70 (2.7%)	2 (0.1%)	<0.001
2017	1,573 (87.6%)	218 (12.1%)	5 (0.3%)	
2018	1,829 (82.5%)	381 (17.2%)	8 (0.4%)	
2019	3,619 (83.6%)	679 (15.7%)	30 (0.7%)	
**Key populations and other risk factors**				***p*****-value**
MSM (b)	1,126 (54.8%)	893 (43.4)	36 (1.8%)	<0.001
PWID (b)	389 (92.6%)	28 (6.7%)	3 (0.7%)	p=0.001
SW (b)	1,790 (86.1%)	274 (13.2%)	16 (0.8%)	<0.001
Reported previous HIV test (b)	5,440 (82.3%)	1,123 (17.0%)	44 (0.7%)	<0.001
Reactive HIV test result on same visit (b)	132 (77.6%)	35 (20.6%)	3 (1.8%)	<0.001
Reported STI last 12 months (b)	405 (76.7%)	110 (20.8%)	13 (2.5%)	<0.001
Reported condomless sex last 12 months (b)	7,179 (87.1%)	1,019 (12.4%)	42 (0.5%)	<0.001
**Knowledge of PEP**				***p*****-value**
Reported knowing PEP (b)	691 (41.0%)	962 (57.0%)	34 (2.0%)	<0.001

*PEP, post-exposure prophylaxis; MTF, male-to-female transgender; FTM, female-to-male transgender; MSM, men who have sex with men; SW, sex workers; PWID, people who inject drugs; STI, sexually transmitted infection*.

*(a)Low prevalence regions include Western Europe (except Portugal), the United States of America, Canada, Oceania, and the Middle East*.

*(b)Showing only results of the respondents reporting the respective category. P-value shown is relative to those not reporting the respective category/reporting the opposite of category shown*.

The reported knowledge of MSM and MTF respondents was higher than that in other key populations. Among MSM, 39.2% reported being aware of PEP, and 45.3% reported being aware of PrEP. Among MTF respondents, 53.6% reported knowledge of PEP and 43.4% reported knowledge of PrEP. In other key populations, reported knowledge was lower. Between 4.5% (Africa) and 19.8% (low prevalence regions) migrants reported knowledge of PEP, whereas, for PrEP, these percentages ranged from 2.3% in African born respondents to 16.8% among those born in low prevalence regions. Natives reported the highest levels of knowledge among all the respondents (23.4% PEP; 20.0% PrEP).

Sex workers reported much lower knowledge levels than MSM and MTF respondents (24.7% PEP; 14.0% PrEP), as did PWID, with even lower percentages (14.5% PEP; 7.4% PrEP). However, as people do not belong exclusively to one key population, there will be an overlap of individuals being included in the different results, individuals who are both MSM and SW or who are both MSM and migrants, for example. Figures highlighting the overlaps in reports of being a member of one or more key populations—among those who report to know PEP and PrEP are available in Supplementary Figure 1. When considering these overlaps, it seems clear that the group most aware of both tools was MSM, either migrants or native.

The reported knowledge of the participants also significantly increased with education, ranging from 6.8% for PEP among those with 9 or less years of formal education to 26.6% among those reporting university level education. For PrEP, the situation was similar, with 3.9% of those reporting no formal education and stating that they knew the tool, whereas 23.6% of those with university level education reported knowing PrEP. Regarding age, again, significant differences were found. The respondents with 50 or more years of age were the least informed about both tools (5.5% know PEP; 3.3% know PrEP). Those 25 or under were most informed about PrEP (16.2%), with those aged 26–49 not far behind (13.8%). For PEP, the situation is reversed in the two age ranges, with those aged 26–49 reporting more knowledge (18.3%) when compared with those 25 or under (16.5%).

Among those previously tested for HIV, 22.1% reported to know PEP, and 17.7% reported to know PrEP. Only 22% of those with a reactive HIV test on the day they responded to the questionnaire reported knowledge of PEP, and 22.4% reported knowledge of PrEP. The situation was similar among those reporting condomless intercourse over the last 12 months (15.8% reported knowing PEP; 12.9% reported knowing PrEP) or an STI in the last 12 months (27.0% reported knowing PEP; 23.3% reported knowing PrEP). Among those reporting to know PrEP, 72.4% also reported knowing PEP, whereas 59.0% of those who reported knowing PEP also reported knowledge of PrEP.

### The Knowledge Level of PEP and PrEP

Table 3 shows the number and percentage of respondents reporting correct, incomplete, or inappropriate knowledge of PEP or PrEP by gender and key population.

**Table 3:** Knowledge levels of PEP and PrEP among the participants reporting to be aware of each tool, by gender and key population.

**PEP**						**PrEP**					
	**Knowledge level**			**Total (n)**	***p*-value**	**Gender**	**Knowledge level**			**Total (n)**	***p*-value**
**Gender**	**Inappropriate knowledge**	**Incomplete knowledge**	**Correct knowledge**				**Inappropriate knowledge**	**Incomplete knowledge**	**Correct knowledge**		
Men	54 (4.7%)	81 (7.1%)	1,010 (88.2%)	1,145	0.211	Men	41 (4.0%)	78 (7.6%)	905 (88.4%)	1,024	0.506
Women	46 (6.3%)	61 (8.4%)	619 (85.2%)	726		Women	15 (5.6%)	16 (6.0%)	237 (88.4%)	268	
MTF	0 (0.0%)	7 (9.6%)	66 (90.4%)	73		MTF	0 (0.0%)	3 (5.4%)	53 (94.6%)	56	
FTM	0 (0.0%)	0 (0.0%)	2 (100.0%)	2		FTM	0 (0.0%)	0 (0.0%)	2 (100.0%)	2	
**Key populations**
Migrants	51 (4.4%)	88 (7.6%)	1,026 (88.1%)	1,165	0.166	Migrants	24 (3.2%)	53 (7.1%)	669 (89.7%)	746	0.158
MSM	33 (3.8%)	58 (6.7%)	771 (89.4%)	862	0.020	MSM	37 (4.1%)	67 (7.4%)	805 (88.6%)	909	0.915
PWID	3 (4.9%)	8 (13.1%)	50 (82.0%)	61	0.265	PWID	1 (3.7%)	0 (0.0%)	26 (96.3%)	27	0.336
SW	30 (5.3%)	50 (8.8%)	490 (86.0%)	570	0.777	SW	11 (4.0%)	18 (6.5%)	246 (89.5%)	275	0.597

*PEP, post-exposure prophylaxis; PrEP, pre-exposure prophylaxis; MTF, male-to-female transgender; FTM, female-to-male transgender; MSM, men who have sex with men; SW, sex workers; PWID, people who inject drugs*.

For both tools, most respondents reporting to be aware of either tool demonstrated correct knowledge, regardless of gender or key population (Table 3). For PEP, 88.2% of men, 85.3% of women, and 90.4% of MTF respondents correctly identified both characteristics of the tool. Conversely, 4.7% of men and 6.3% of women did not identify either. The two FTM participants who responded to the question correctly identified both statements. The situation was similar when analyzing by key population, with over 80% of respondents from all four key populations correctly reporting both statements (Table 3). Incorrect knowledge was also low within each key population, with the lowest being among MSM (3.8%) and the highest among SW (5.3%).

For PrEP, the data were similar, with 88.4% of men and women and 94.6% of MTF respondents correctly identifying both tools. Again, the two FTM respondents had correct knowledge of PrEP. Inappropriate knowledge was also low and was only observed in men (4.0%) and women (5.6%). The analysis by key population revealed a similar scenario, with 89.7% of migrants, 88.6% of MSM, 96.3% of PWID, and 89.5% of SW reporting both statements. Inappropriate knowledge by the key population ranged from 3.2% among migrants to 4.1% among MSM (Table 3).

### Factors Associated With Knowledge of PEP and PrEP

Tables 4, 5 present the results of the univariate and multivariate logistic regressions, Table 4 for knowledge of PEP, and Table 5 for knowledge of PrEP.

**Table 4:** Factors associated with knowledge of PEP.

	**OR (95% C.I.)**	***p*-value**	**AOR (95% C.I.) (c)**	***p*-value**
**Country or region of birth**				
Portugal	Reference category
Low prevalence regions (a)	0.81 (0.68–0.96)	0.014	0.59 (0.49–0.71)	<0.001
South America	0.89 (0.80–0.99)	0.032	0.81 (0.71–0.91)	<0.001
Africa	0.15 (0.13–0.18)	<0.001	0.23 (0.19–0.27)	<0.001
Eastern Europe and Asia	0.31 (0.23–0.43)	<0.001	0.28 (0.20–0.38)	<0.001
**Gender**				
Male	Reference category
Female	0.67 (0.60–0.74)	<0.001	0.77 (0.69–0.86)	<0.001
MTF	5.36 (3.81–7.54)	<0.001	5.00 (3.48–7.18)	<0.001
FTM	1.99 (0.51–7.70)	0.320	1.60 (0.40–6.41)	0.500
**Age**				
≤ 25 years	Reference category
26–49 years	1.14 (1.02–1.27)	0.02	1.23 (1.10–1.39)	<0.001
≥ 50 years	0.30 (0.24–0.36)	<0.001	0.60 (0.48–0.75)	<0.001
**Education**				
Basic (≤ 9 years)	Reference category
Secondary (12 years)	2.86 (2.48–3.29)	<0.001	2.14 (1.84–2.49)	<0.001
University (Bachelors or higher)	5.08 (4.43–5.84)	<0.001	3.69 (3.18–4.29)	<0.001
**Test year**				
2016	Reference category
2017	2.92 (2.45–3.49)	<0.001	2.05 (1.70–2.48)	<0.001
2018	3.18 (2.69–3.77)	<0.001	1.88 (1.56–2.25)	<0.001
2019	2.47 (2.10–2.91)	<0.001	1.59 (1.33–1.89)	<0.001
**Key populations and other risk factors**				
MSM (b)	5.38 (4.85–5.97)	<0.001	5.24 (4.47–6.14)	<0.001
PWID (b)	0.89 (0.67–1.16)	0.893	0.94 (0.70–1.26)	0.680
SW (b)	2.26 (02.02–2.53)	<0.001	2.46 (2.13–2.84)	<0.001
Previous HIV test (b)	4.31 (3.78–4.91)	<0.001	3.67 (3.19–4.22)	<0.001
STi in last 12 months (b)	2.24 (1.86–2.71)	<0.001	1.73 (1.41–2.12)	<0.001
Condomless IC last 12 months (b)	0.66 (0.59–0.74)	<0.001	0.73 (0.64–0.82)	<0.001
Reactive HIV test result in same visit (b)	1.56 (1.12–2.19)	0.100	1.24 (0.84–1.84)	0.275
KnowsPrEP (b)	33.05 (28.70–38.07)	<0.001	24.39 (20.85–28.53)	<0.001

*OR, crude odds ratio; C.I., confidence interval; AOR, adjusted odds ratio; MTF, male-to-female transgender; FTM, female-to-male transgender; MSM, men who have sex with men; SW, sex workers; PWID, people who inject drugs*.

*(a)Low prevalence regions include Western Europe (except Portugal), the United States of America, Canada, Oceania, and the Middle East*.

*(b)Reported OR is relative to responding “no” to the respective category, or not belonging to the corresponding key population*.

*(c)Adjusted for country or region of birth, gender, age, and education. In the corresponding categories, adjusted for the three remaining variables (e.g., gender is adjusted for country or region of birth, age, and education)*.

**Table 5:** Factors associated with knowledge of PrEP.

	**OR (95% C.I.)**	***p*-value**	**AOR (95% C.I.) (c)**	***p*-value**
**Country or region of birth**				
Portugal	Reference category
Low prevalence Regions (a)	0.79 (0.65–0.97)	0.023	0.63 (0.50–0.78)	<0.001
South America	0.79 (0.69–0.89)	<0.001	0.98 (0.85–1.13)	0.789
Africa	0.09 (0.07–0.12)	<0.001	0.17 (0.13–0.22)	<0.001
Eastern Europe and Asia	0.33 (0.23–0.47)	<0.001	0.34 (0.24–0.49)	<0.001
**Gender**				
Male	Reference category
Female	0.27 (0.24–0.31)	<0.001	0.29 (0.25–0.34)	<0.001
MTF	3.56 (2.49–05.08)	<0.001	3.27 (2.22–4.81)	<0.001
FTM	1.29 (0.27–6.22)	0.752	0.93 (0.18–4.72)	0.927
**Age**				
≤ 25 years	Reference category
26–49 years	0.83 (0.74–0.94)	0.003	0.86 (0.75–0.99)	0.032
≥ 50 years	0.17 (0.13–0.23)	<0.001	0.39 (0.29–0.53)	<0.001
**Education**				
Basic (≤ 9 years)	Reference category
Secondary (12 years)	3.98 (3.30–4.82)	<0.001	2.76 (2.26–3.38)	<0.001
University (Bachelors or higher)	7.79 (6.47–9.38)	<0.001	5.25 (4.30–6.41)	<0.001
**Test Year**				
2016	Reference category
2017	4.99 (3.80–6.57)	<0.001	2.85 (2.14–3.81)	<0.001
2018	7.51 (5.80–9.73)	<0.001	3.87 (2.94–05.09)	<0.001
2019	6.97 (5.44–8.93)	<0.001	4.52 (3.48–5.88)	<0.001
**Key populations and other risk factors**				
MSM (b)	14.98 (13.18–17.02)	<0.001	15.48 (12.46–19.23)	<0.001
PWID (b)	0.53 (0.36–0.76)	<0.001	0.62 (0.42–0.94)	0.023
SW (b)	1.45 (1.25–1.68)	<0.001	2.03 (1.68–2.46)	<0.001
Previous HIV test (b)	3.85 (3.31–4.49)	<0.001	3.68 (3.11–4.36)	<0.001
STi in last 12 months (b)	2.88 (2.32–3.57)	<0.001	2.29 (1.81–2.90)	<0.001
Condomless IC last 12 months (b)	0.68 (0.59–0.78)	<0.001	0.75 (0.65–0.88)	<0.001
Reactive HIV test result in same visit (b)	2.09 (1.45–3.02)	<0.001	1.48 (0.97–2.27)	0.072
Knows PEP	33.05 (28.70–38.07)	<0.001	23.73 (20.29–27.75)	<0.001

*OR, crude odds ratio; C.I., confidence interval; AOR, adjusted odds ratio; MTF, male-to-female transgender; FTM, female-to-male transgender; MSM, men who have sex with men; SW, sex workers; PWID, people who inject drugs*.

*(a)Low prevalence regions include Western Europe (except Portugal), the United States of America, Canada, Oceania, and the Middle East*.

*(b)Reported OR is relative to responding “no” to the respective category, or not belonging to the corresponding key population*.

*(c)Adjusted for country or region of birth, gender, age, and education. In the corresponding categories, adjusted for the three remaining variables (e.g., gender is adjusted for country or region of birth, age, and education)*.

Being born outside Portugal was associated with lower knowledge of PEP, with the knowledge of those born in South America being the most similar to natives (AOR.81; 95% CI.71–0.91). Compared with males, being a MTF was associated with higher knowledge (AOR 5.00; 95% CI 3.48–7.18) and being a female was associated with lower knowledge (AOR 0.77; 95% CI.69–0.86). Being 25–49 years of age (AOR 1.23; 95% CI 1.10–1.39), compared with those under 25; being tested for the first time in the network after 2016 (Table 4); and having secondary (AOR 2.14; 95% CI 1.84–2.49) or university level education (AOR 3.69; 95% CI 3.18–4.29) were also significantly associated with increases in knowledge.

Identifying as belonging to MSM (AOR 5.24; 95% CI 4.47–6.14) or SW (AOR 2.46; 95% CI 2.13–2.84) was also associated with increased knowledge of PEP, as was reporting an STI diagnosis in the last 12 months (AOR 1.73; 95% CI 1.41–2.12), reporting having had a previous HIV test (AOR 3.67; 95% CI 3.19–4.22), and reporting to know PrEP (AOR 24.39; 95% CI 20.85–28.53). Reporting condomless intercourse in the last 12 months was associated with lower levels of knowledge of PEP (AOR 0.73; 95% CI 0.64–0.82).

The situation for PrEP is quite similar, with multiple factors showing significant associations with reported knowledge (Table 5). These include country or region of birth, where, again, being a Portuguese national is associated with higher levels of reported knowledge (Table 5); gender, where, again, the MTF respondents were much more likely to know PrEP (AOR 3.27; 95% CI 2.22–4.81) and women were less likely to know PrEP (OR.29; 95% CI.25–0.34) when compared with men. Those aged 25 or younger are more likely to know PrEP than those aged 26–49 (AOR.86; 95% CI.75–0.99), and that those aged 50 or above (AOR.39; 95% CI.29–0.53) and those being tested for the first time in the network in more recent years were associated with higher levels of PrEP knowledge (Table 4).

Formal education was also associated with higher levels of knowledge of PrEP. Secondary education (AOR 2.76; 95% CI 2.26–3.38) and university-level education (AOR 5.25; 95% CI 4.30–6.41) were both significantly associated with higher knowledge. Regarding key populations, identifying as belonging to MSM (AOR 15.48; 95% CI 12.46–19.23) or SW (AOR 2.03; 95% CI 1.68–2.46) was associated with increases in knowledge, whereas identifying as belonging to PWID was negatively associated with knowing PrEP (AOR 0.62; 95% CI 0.42–0.94). Additionally, and similarly to PEP, reporting a previous HIV test (AOR 3.68; 95% CI 3.11–4.36), an STI in the last 12 months (AOR 2.29; 95% CI 1.81–2.90), and reporting to know PEP (AOR 23.73; 95% CI 20.29–27.75) were also associated with knowing PrEP. Lastly, those reporting condomless intercourse in the last 12 months were less likely to be informed about PrEP (AOR 0.75; 95% CI.65–0.88).

### Use of PEP and PrEP

The ue of PEP and PrEP, in general, was extremely low in the sample, with 1.8% of respondents reporting to have used PEP (Table 1) and.4% reporting to have used PrEP (Table 2). Based on gender, the reported percentage of PEP use was higher among MTF (6.5%), followed by men (2.2%). Only 1.2% of women reported to have used PEP, and 10% of female-to-male transgender (FTM) respondents reported to have used this tool (1/10 respondents). The respondents born in South America (3.5%) and Portugal (2.0%) had the highest reported percentages of PEP use, with those born in Africa reporting the lowest use (0.4%).

Pre-exposure prophylaxis use was higher in those aged 26–49 (2.3%), with 1.5% among the next highest age range, those 25 or younger. Based on education level, which is similar to knowledge, PEP use is reported more frequently by those with higher levels of formal education. Only.4% of those with <9 years of formal education reported use of PEP, going up to 3.1% among those with university-level education.

MSM was the key population where the use of PEP was highest, with 5.4% of MSM reporting the use of PEP. SW followed MSM as the second highest, with 3.2% reporting the use of this tool. The use of PEP was also reported by 2.9% of those with a previous HIV test, by 4.0% of those with a reactive HIV test, and by 3.7% of those reporting an STI, whereas only 1.8% of those reporting condomless sex. Both the overall report of PEP use in absolute numbers, and the percentage of respondents reporting its use (0.7% in 2016 to 2.1% in 2019) increased over time (Table 1).

As for PrEP, use was concentrated in men, although only.7% of men reported using PrEP. The percentage of all genders reporting PrEP use was low:0.1% for women; 2.3% for MTF; 0% in FTM respondents (although only nine FTM respondents overall). Regarding country or region of birth, Portugal and South America represented 88.9% of PrEP users, and those aged 26–49 account for 75.6%. MSM accounted for 80% of PrEP users, whereas PWID accounted for only 6.7%, and SW accounted for 35.6%. Almost the entirety of PrEP users had a previous HIV test (97.8%), and 88.9% of those who reported to have used PrEP had a negative HIV test result on the day they responded to the questionnaire. PrEP users were also more frequently found among those reporting no STI in the last 12 months (53.3%) and among those reporting condomless intercourse in the last 12 months (93.3%). Lastly, the absolute number of persons reporting to have used PrEP increased over the 4 years, but its maximum number was 30 in 2019, rising from 8 in 2018.

## Discussion

This study is the first to look at PEP and PrEP knowledge and use outside the MSM community and adds to the body of evidence of these subjects in Europe. Low percentages of knowledge and use of both prevention tools were also found among key populations in both the United States (20) and China (21), although the use of both tools in our sample is lower than that reported in those studies.

Specifically, regarding PEP knowledge and uptake among MSM, our results were lower than the pooled proportion found in a recent systematic review (22). Similar to other research conducted in Italy, PEP awareness in this group in our sample was associated with younger ages, higher education, and previous HIV test. Other factors linked to higher awareness included factors not assessed in this study, such as the level of HIV stigma and more frequent contact with HIV/AIDS organizations (23).

The use of PEP and PrEP among MSM was higher than those reported in a large Europe-wide study (24), where 4.5% of MSM had notpreviously tested for HIV or had a negative test result for HIV or 3% with a reactive test result for HIV). In the same study, 3.3% of those who never tested or with a negative test and 1.2% of those with a reactive test reported PrEP use, whereas, in our sample, the reported percentage of MSM who report to have taken PrEP is 1.8%. EMIS data were collected in 2017, when PrEP availability in Europe was much lower than it currently is (25, 26), which may explain the higher percentage of reported users in Portugal, compared with those with a reactive test in the EMIS sample.

This study also finds a lower percentage of PrEP use among MSM than that found in a cohort of MSM in Lisbon, where 3.2% of the participants reported PrEP use (27), indicating that our sample will likely represent another subset of MSM, with less access or willingness to take PrEP. The low knowledge of SW regarding PrEP (28) is similar to what was found in other research conducted in the US with street-based SW and in China (29). Data on PEP in this group are extremely scarce (30). With proven acceptability of PrEP among female SW (31–33) and no evidence of risk compensation (34), this suggests ample space to scale up access to both prevention tools in the country among this group and underlines the need for further research into awareness and use of both tools among sex worker communities.

As for PWID, which present some of the lowest percentages of knowledge and use in the sample, little attention has been given to PEP and PrEP knowledge and use in this community (35). A recent systematic review (36) of PrEP in this group revealed high awareness but low usage rates, ranging from 0 to 3%. The low knowledge levels of PrEP and PEP found among PWID in this study suggest a need to invest in increasing awareness of the tool among this group, and the level of use reinforces the urgency of adequately including PWID in PrEP programs, as well as information and ease of access to PEP.

In terms of reported knowledge by gender, MTF transgender individuals were the only exception to overall very low knowledge of PEP, with over 50% of the sample knowing this tool. Despite this group reporting the highest levels of awareness and use of both tools, they are lower than those reported in other studies, conducted in high-income settings (37, 38), particularly with regard to their use. Although this may reflect a greater investment in disseminating information about these tools among MTF individuals in the country, particularly those linked to commercial sex work, reported use in this study suggests ample space to scale up this prevention tool among transgender women, who continue to carry a disproportionate burden of HIV globally (39).

Lower levels of knowledge in and use of PrEP by women are in line with existing literature (29), despite recent European data showing the interest of women who are at high risk of acquiring HIV in accessing PrEP (40), underlining the relevance of a targeted information strategy to reach women at high risk.

Concerning region of birth, African-born respondents reported much lower levels of knowledge and use of both tools than other regions. As the continent still carries the highest burden of HIV globally, and that a high percentage of new HIV diagnosis in Portugal in recent years was among people born in African countries, especially Portuguese-speaking African countries (2), increasing knowledge and access, with a view to increasing its use in these communities, could lead to HIV reduction in the country.

Improving communication strategies to reach all the key populations analyzed here, with information on these tools, which is adjusted to their needs and education levels, is paramount, particularly considering that reported knowledge was very low among the persons who could have directly benefited from these tools, specifically those with a reactive HIV test result in the same visit (22% PEP; 22.4% PrEP), reporting condomless sexual intercourse in the last 12 months (15.8% PEP; 12.9% PrEP), or reporting an STI in the last 12 months, with syphilis diagnosis being a documented predictor of HIV infections among at least MSM in the country (41).

This study has several limitations, including the self-reported nature of all the data collected, the fact that the sample is composed of persons who were tested in community-based centers in the country, and the sample will not be representative of the key populations analyzed, despite the high number of respondents included. Within those being tested in the community-based centers, our study only included those responding to the PEP and PrEP questions, who will likely represent persons more interested in HIV prevention; therefore, the overall knowledge and use could be even lower among all the users. Additionally, the study did not investigate willingness to use these tools, which can be an important next step, or into barriers to access, which will, no doubt, condition the low levels of use reported. The regression model may also miss the key factors associated with knowledge of these prevention tools, as they were not part of the questionnaire.

## Conclusions

Our results show that key populations, most at risk for HIV, still have meaningful gaps in terms of knowledge of available prevention tools. Limited knowledge of both tools and recent HIV cases represents missed opportunities to improve knowledge and access to PEP and PrEP.

Increasing available information and effective access to PEP, including through clear guidance on mechanisms to access this tool and inclusion of underserved key populations in PrEP programs, is paramount to break transmission chains both within and outside the MSM community, considering that, in Portugal, HIV remains concentrated in the key populations.

An effort to systematically provide an integrated approach to counseling where ARV drugs are included as a prevention measure at all HIV testing sites and, particularly, at community-based testing services working with any of the concerned key populations, is recommended, given the high number of the persons reporting previous HIV tests who were still unaware of these preventive tools.

Future research should assess both preferences and efficiency of strategies to increase knowledge of these tools, including through digital platforms when possible, to inform public health interventions to bridge this knowledge gap. Additionally, given the small body of evidence available regarding knowledge and the use of PEP and PrEP among the key populations, research that investigates both these dimensions in other countries is encouraged to identify knowledge gaps and access barriers. Lastly, research on the willingness to use these tools among individuals at a high risk of infection and factors hindering their ability to access them would be desirable.

## Data Availability Statement

The raw data supporting the conclusions of this article will be made available by the authors upon approval of a written proposal detailing the data required and its purpose.

## Ethics Statement

This study was reviewed and approved by the Ethics Committee of Instituto de Saúde Pública da Universidade do Porto. Written informed consent from the participants' legal guardian/next of kin was not required to participate in this study in accordance with the national legislation and the institutional requirements. Oral informed consent is requested to all participants before responding.

## Author Contributions

DS developed the first draft with support from PM and MR, and supervision from HB. DS and PM conducted the statistical analysis. All authors provided input to the draft versions of the manuscript and validated the final version.

## Conflict of Interest

The authors declare that the research was conducted in the absence of any commercial or financial relationships that could be construed as a potential conflict of interest.

## Publisher's Note

All claims expressed in this article are solely those of the authors and do not necessarily represent those of their affiliated organizations, or those of the publisher, the editors and the reviewers. Any product that may be evaluated in this article, or claim that may be made by its manufacturer, is not guaranteed or endorsed by the publisher.
