# Supplementary material for: Knowledge and Use of PEP and PrEP Among Key Populations Tested in Community Centers in Portugal
Source: Front Public Health. 2021 Jul 23;9:673959. doi: 10.3389/fpubh.2021.673959 (PMC8342856; doi:10.3389/fpubh.2021.673959)
Supplement: Supplementary file 1 [file Table_1.DOCX]

**Supplementary Figure 1.** Overlaps in reports of being a member of one or more key populations, among those reporting to know PEP or PrEP


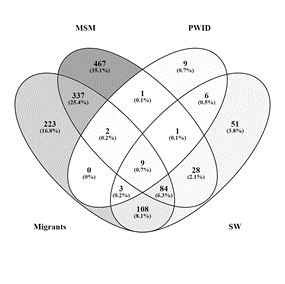


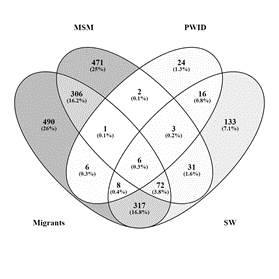


1. Reported knowing PEP (B) Reported knowing PrEP

Tone of grey indicates the relative percentage of respondents in each intersection. Darker tones indicate higher relative percentages.
