# Supplementary material for: Knowledge and Use of PEP and PrEP Among Key Populations Tested in Community Centers in Portugal
Source: Front Public Health. 2021 Jul 23;9:673959. doi: 10.3389/fpubh.2021.673959 (PMC8342856; doi:10.3389/fpubh.2021.673959)
Supplement: Supplementary file 2 [file Table_2.DOCX]

**Supplementary Table 1.** Characteristics of overall baseline respondents, PEP respondents and PrEP respondents

|  | **Baseline** | **PEP respondents** | **PrEP respondents** |
| --- | --- | --- | --- |
| **Total questionnaires** | 53809 | 12983 | 10973 |
|  | n (%) | n (%) | n (%) |
| **Age (a)** | | | |
| ≤ 25 years | 13166 (24.5) | 3338 (25.9) | 2889 (26.3) |
| 26-49 years | 25550 (47.5) | 7368 (57.1) | 6231 (56.8) |
| ≥ 50 years | 15027 (27.9) | 2173 (16.9) | 1839 (16.8) |
| **Gender** | | | |
| Male | 26852 (49.9) | 6691 (51.9) | 5830 (53.1) |
| Female | 26754 (49.7) | 6054 (47.0) | 5005 (45.6) |
| Transgender MTF | 168 (0.3) | 138 (1.1) | 129 (1.2) |
| Transgender FTM | 35 (0.1) | 10 (0.1) | 9 (0.1) |
| **Country of birth (a)** | | | |
| Portugal | 27801 (51.7) | 3489 (27.1) | 3129 (28.5) |
| Low prevalence regions | 1728 (3.2) | 1017 (7.9) | 834 (7.6) |
| Africa | 14477 (26.9) | 4217 (32.7) | 3406 (31.0) |
| South America | 6993 (13.0) | 3611 (28.0) | 3134 (28.6) |
| Eastern Europe and Asia | 1417 (2.6) | 543 (4.2) | 455 (4.1) |
| **Highest completed education level (a)** | | | |
| Basic (≤ 9 years) | 22317 (41.5) | 4535 (35.2) | 3833 (34.9) |
| Secondary (12 years) | 14441 (26.8) | 4387 (34.0) | 3739 (34.1) |
| University (Bachelors or higher) | 9674 (18.0) | 3490 (27.1) | 2990 (27.2) |
| **Previously tested for HIV (a)** | | | |
| No | 23313 (43.3) | 4664 (36.2) | 3997 (36.4) |
| Yes | 26111 (48.5) | 7771 (60.3) | 6607 (60.2) |
| **STI in previous 12 months (a)** | | | |
| No | 41979 (95.9) | 10481 (81.3) | 8839 (80.6) |
| Yes | 1796 (4.1) | 593 (4.6) | 528 (4.8) |
| **Condomless intercourse previous 12 months (a)** | | | |
| No | 5918 (13.7) | 1998 (15.5) | 1751 (16.0) |
| Yes | 37343 (86.3) | 9702 (75.3) | 8240 (75.1) |
| **HIV Test result in the same visit (b)** | | | |
| Non reactive | 51849 (99.0) | 12247 (95.0) | 10399 (94.8) |
| Reactive | 508 (1.0) | 200 (1.6) | 170 (1.5) |
| **Key Populations** | | | |
| MSM | 3463 (6.4) | 2275 (17.6) | 2055 (18.7) |
| SW | 3428 (6.4) | 2373 (18.4) | 1080 (19.0) |
| PWID | 1303 (2.4) | 456 (3.5) | 420 (3.8) |

(a) Remaining values and percentages are missing values in the questionnaires

(b) Missing individuals did not perform an HIV test
